# The disappearance of the “revolving door” patient in Scottish general practice: successful policies

**DOI:** 10.1186/1471-2296-13-95

**Published:** 2012-10-04

**Authors:** Andrea E Williamson, Paul CD Johnson, Kenneth Mullen, Philip Wilson

**Affiliations:** 1 General Practice and Primary Care, College of MVLS, University of Glasgow, 1 Horselethill Rd, Glasgow, G12 ORR, UK; 2 Robertson Centre for Biostatistics, Institute of Health and Wellbeing, College of MVLS, University Avenue, University of Glasgow, Glasgow, G12 8QQ, UK; 3 School of Medicine, College of MVLS, University of Glasgow, Gartnavel Royal Hospital, 1055 Great Western Road, Glasgow, G12 0XH, UK; 4 University of Aberdeen, The Centre for Health Science, Old Perth Road, Inverness, IV2 3JH, UK

## Abstract

**Background:**

We describe the health of "revolving door" patients in general practice in Scotland, estimate changes in their number over the timescale of the study, and explore reasons for changes, particularly related to NHS and government policy.

**Methods:**

A mixed methods predominantly qualitative study, using a grounded theory approach, set in Scottish general practice. Semi-structured interviews were conducted with professional key informants, 6 Practitioner Services staff who administer the GP registration system and 6 GPs with managerial or clinical experience of working with “revolving door” patients. Descriptive statistical analysis and qualitative analysis of patient removal episodes linked with routine hospital admissions, outpatient appointments, drug misuse treatment episodes and deaths were carried out with cohorts of “revolving door” patients identified from 1999 to 2005 in Scotland.

**Results:**

A “revolving door” patient is removed 4 or more times from GP lists in 7 years. Patients had complex health issues including substance misuse, psychiatric and physical health problems and were at high risk of dying. There was a dramatic reduction in the number of “revolving door” patients during the course of the study.

**Conclusions:**

“Revolving door” patients in general practice had significant health problems. Their numbers have reduced dramatically since 2004 and this probably resulted from improved drug treatment services, pressure from professional bodies to reduce patient removals and the positive ethical regulatory and financial climate of the 2004 GMS GP contract. This is a positive development for the NHS.

## Background

Interest in “revolving door” patients, that is those who are repeatedly removed from successive general practice (GP) lists, arose from pilot work on how patients achieve registration with practices in the city of Glasgow. General practices in the United Kingdom operate a geographical list system which defines their patient population. Being registered with a general practitioner (on a list) is necessary to access most National Health Service (NHS) facilities. We wished to find out more about this group of patients who appeared to be systematically excluded from a health system that is internationally lauded as providing trust, coordination, continuity, flexibility and coverage, irrespective of health status or ability to pay [1].

General practices in the UK have a right to remove patients from their list for a variety of reasons including “break down in the doctor-patient relationship” or “violence”. “Moved out of the practice area,” is more commonly used but this category is rarely relevant when considering “revolving door” patients. There is a literature on patients who have been removed once from GP lists but it has either excluded repeatedly-removed patients from analysis [2] or has recommended management strategies without any consideration of their characteristics or the reasons for their repeated removal [3].

In this paper we develop a definition of “revolving door” patients in general practice and describe their health. We also report on changes in the number of “revolving door” patients in Scotland over the timescale of the study and the possible NHS and government policy reasons for this. The explanations as to why patients may become “revolving door” patients and the implications for our understanding of doctor-patients relationships will be considered in a future paper.

## Methods

In 2006, six semi-structured interviews were carried out with Practitioner Services staff who administered the GP registration system across Scotland and with two GPs whose managerial or clinical role meant they had worked with “revolving door” patients. Data from these interviews guided the development of a definition of a “revolving door” patient and this definition was applied to the patient removal data (for reasons other than change of address) obtained from the Community Health Index (CHI) from 1999 to 2005. Secondary care data on hospital admissions, outpatient attendances and drug misuse treatment episodes experienced over the life course were linked by NHS Information Services Division (ISD) Scotland to a sample. The results for the adults are presented in this paper. Additional file 1: Appendix 1 describes the statistical analysis in detail.

Drawing on cognitive psychology script theory about clinical decision making [4], the sensitizing concept (idea that helps to shape theory generation) [5] of “patient scripts” was used to categorize patients’ predominant health issues into categories that described the cohort overall and also guided the direction of statistical analysis. Additional file 1: Appendix 2 describes the use of “patient scripts” to do this.

A further four semi-structured interviews were conducted in 2010 with GPs in health board areas where “revolving door” patient prevalence was high. Summaries of patient removal data from ISD Scotland (1999 to 2011) were also reviewed and analyzed.

Our predominantly qualitative analysis using mixed methods was informed by the grounded theory approach attributable to Charmaz [5]. The results were integrated in a dialectic way; that is they were compared to seek and explain differences between them [6].

## Results

### Definition

“Revolving door” patients in general practice were described by the professional key informants in 2006 as a small group of patients that professionals working in primary care would recognise. In 2010 one GP described coming across 20–30 “revolving door” patients during a 15 year partnership in an urban area that used to generate a lot of repeat removals (GP respondent 3, (GP3)).

In 2006 Practitioner Services respondents agreed that a patient who had been removed once or twice was not a “revolving door” patient, but three removal episodes was “starting to look like a problem”. There was a range of views on removal frequency. Respondents made a distinction between two groups of “revolving door” patients; “fast revolvers” who were regularly and routinely removed as frequently as every seven days and “slow revolvers” who were repeatedly removed, but after months rather than days or weeks.

We developed several versions of the definition in an attempt to describe these qualitatively derived categories so included all patients removed four or more times during the study interval. We tested categories based on the median number of days patients spent on a GP list. Those with a median of 0–100 days on list were “fast revolving door” patients, 101–180 were “medium revolving door” patients and those with medians of more than 181 days we called “slow revolving door” patients.

Table 1 shows there were no substantial differences in demographic characteristics (sex, age, marital status and deprivation) between these categories, although the proportion of males was slightly lower among patients who had stayed on a GP list with a median of more than 180 days compared with faster revolving patients (59% v 69%; P = 0.045). Additional file 2: Appendix 3 summarises additional tests that we carried out across the data to compare the “fast”, “medium” and “slow” revolving door categories. We could find no additional statistical differences between the groups from these tests either.

**Table 1:** Sex, age, marital status and Scottish Index of Multiple Deprivation (SIMD) 2006 decile of the 555 “revolving door” patients, overall and compared between subgroups defined by median days on a practice list

		**Total**	**Median days on GP list**			**P**^**a**^	**P**
			**Fast (0–100)**	**Medium (101–180)**	**Slow (181+)**	**Slow vs rest**	**Fast vs medium**
Sex	N_OBS_ (N_MISSING_)	555 (0)	309 (0)	113 (0)	133 (0)	0.045	0.342
	N (%) male	371 (66.8%)	218 (70.6%)	74 (65.5%)	79 (59.4%)		
Age (years) at first removal	N_OBS_ (N_MISSING_)	555 (0)	309 (0)	113 (0)	133 (0)	0.163	0.679
	Mean (SD)	34 (13)	34 (13)	35 (14)	32 (11)		
Married at first removal	N_OBS_ (N_MISSING_)	392 (163)	135 (17)	112 (28)	145 (118)	0.474	0.178
	N (%) married	62 (15.8%)	27 (20.0%)	15 (13.4%)	20 (13.8%)		
SIMD decile at first removal [1]	N_OBS_ (N_MISSING_)	409 (146)	271 (38)	77 (36)	61 (72)	0.112	0.140
	Median (IQR)	9.0 (7.0, 10.0)	9.0 (7.0, 10.0)	8.0 (6.0, 10.0)	9.0 (7.0, 10.0)		

^a^P-values are from Wilcoxon rank sum tests (age and SIMD decile) and Fisher exact tests (sex and married status).

^b^Reporting convention in SIMD 2006, 10= most deprived decile.

Because there was no difference between these groups our final working definition of a “revolving door” patient was a patient who was removed four or more times from a GP practice list in seven years.

There were demographic descriptive data for 555 adult “revolving door” patients, and health service linked data for 410 patients. 351 patients were included in the qualitative analysis. Additional file 2: Appendix 4 summarises the three samples of “revolving door” patients. They were considered representative of the “revolving door” patient cohort as a whole.

Most (83%) “revolving door” patients were removed between four and seven times (range 4–92), and most (67%) were male. The median age at first removal was 31 years (range 17–88). Thirty-five percent of “revolving door” patients lived in the most deprived decile and 87% in the more deprived half of Scotland (decile 6–10) [7]. Eighty-four percent were not married at their first removal (compared with 51% of the general Scottish population aged 30–34) [8].

### Morbidity

Eighty-six percent of the 410 record-linked “revolving door” patients had at least one hospital admission (median 9 admissions, range 0 to 295) before 2011. The reasons for admission are summarized in Table 2.

**Table 2:** Percentage of the 410 record-linked “revolving door” patients with at least one hospital admission, by health problem

**Percentage of cohort**	**Reason for at least one admission**
78%	physical health problem
68%	substance misuse problem
52%	poisoning
49%	intervention or procedure
39%	victim of violence
38%	psychiatric illness
78%	symptom only eg chest pain, collapse

Fifty-one percent had taken an irregular discharge during a hospital admission (median 1, range 0 to 45). Ninety-nine percent had had a hospital outpatient appointment (median 15, range 0 to 249) and 92% of patients had missed outpatient appointments (median 7, range 0 to 146).

There were no substantial correlations between numbers of removal episodes and other patterns of health service activity, including irregular discharges (Spearman’s ρ = 0.02, P = 0.743) and number of admissions (Spearman’s ρ = 0.07, P = 0.170). There was however a weak positive correlation with the number of outpatients appointments (Spearman’s ρ = 0.11, P = 0.032), although not with the number of missed outpatients appointments (Spearman’s ρ = 0.07, P = 0.141). There was a tendency for hospital admission dates to be close to removal dates (odds = 1.25, P = 0.008) but not to re-registration dates (odds = 1.16, P = 0.084) than could be expected by chance (a health service date was defined as close to a practice transfer date if fell in the first or last 25% of the period between two practice transfer dates; see statistical methods). Dates of drug treatment episodes were close to both removal (odds = 1.26, P = 0.049) and registration dates (odds = 2.05, P < 0.001). Outpatient appointment dates were not associated with removal (odds = 0.98, P = 0.740) or registration dates (odds = 1.03, P = 0.701).

From all the collated data sources it was determined that 84% of patients had been dependent on substances. The majority had evidence of dependency on opiates but patients with alcohol dependency were also numerous. Alcohol-related admissions were important for many patients in the cohort.

Forty-eight percent of the cohort had evidence of self harm and 18% had a definite personality disorder diagnosis. There was a low prevalence of ‘severe and enduring’ (chronic psychotic or severe mood disorders) mental health problems.

Qualitative analysis of the “revolving door” patients gives an overview of the main health problems of the sample and is summarized in Table 3 together with examples of patient profiles in each category. Patient profiles which were coded “no clinical code possible” had insufficient evidence from the linked secondary care and drug misuse data to be able to determine their main health problems. We did not have access to primary care records.

**Table 3:** Predominant health problems from the qualitative analysis of “revolving door” patients with typical examples of patient profiles

**Predominant health code “patient script”**	**% of patients**	**Patient profile examples**
**Substance misuse and psychiatric illness**	**18%**	Female patient in her 50s, 300 admissions. Shifting psychiatry diagnoses; depression, anxiety, with personality disorder, self harm and alcohol dependency. Drug misuse treatment episodes for opiate dependency, and additional physical health problems, long term neurological condition and epilepsy. Missed 3/23 outpatient appointments. Removed 5 times from GP lists.
**Drug dependency problems**	**15%**	Male patient in his 40s, 20 admissions. Opiate dependent with drug misuse treatment episodes, admissions with recurrent cutaneous abscesses, chronic hepatitis C infection and occasionally asthma. Missed 11/14 outpatient appointments. Removed 22 times from GP lists.
**Psychiatric illness and physical illness**	**10%**	Male patient in his 40s, 80 admissions. Sporadic diagnosis of conduct disorder, evidence of self harm, and was alcohol dependent. Many admissions due to disability after major trauma. Missed 1/3 outpatient appointments. Removed 4 times from GP lists.
**Substance misuse and physical illness**	**6%**	Male patient in his 50s, 9 admissions. History of malignant disease and was alcohol dependent with physical complications of alcohol dependency. Had no outpatient appointments. Removed 10 times from GP lists.
**Alcohol related harm**	**7%**	Female patient in her 60s, 60 admissions. Alcohol dependent and who had alcohol related brain injury, seizures and alcoholic liver disease. Missed 2/13 outpatient appointments. Removed 4 times from GP lists.
**Psychiatric illness**	**6%**	Male patient in his 30s, 65 admissions. Several admissions with diagnosis of paranoid schizophrenia, various personality disorder diagnoses, admissions following medicines and opiate overdoses, drug dependence and alcohol dependence and some physical consequences of drug use starting to become apparent. Missed 12/24 outpatient appointments. Removed 8 times from GP lists.
**Injuries**	**5%**	Male patient in his 30s, 10 admissions. Contusions of the thorax, lower back and pelvis, pneumothorax, scalp wound injury, open wounds of abdomen lower back and pelvis, drug dependency, evidence of self harm and asthma. Missed 16/38 outpatient appointments. Removed 4 times from GP lists.
**Physical illness**	**4%**	Female patient in 20s, 5 admissions. She had nausea and vomiting, biochemical and coagulation problems. The underlying diagnosis was unclear. Missed 5/6 outpatient appointments including psychiatry appointments. Removed 5 times from GP lists.
**No clinical code possible**	**29%**	Male patient in his 30s, 10 admissions. Open wound to forearm and no other recorded information on other admissions. 5 outpatient appointments in oral surgery, orthopaedics and ENT with 1/5 missed appointments. Removed 5 times from GP lists.

### Mortality

**Figure 1:**
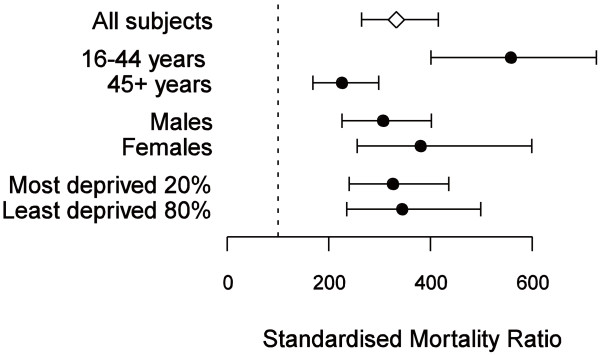
**Estimates of the standardised mortality ratio (SMR; the number of deaths observed per 100 expected) among the 410 record-linked “revolving door” patients relative to the general Scottish population in 2004, overall and in subgroups of age, sex and deprivation.** Error bars represent 95% confidence intervals. The SMR estimates were adjusted for differences between the “revolving door” cohort and general Scottish population in the distributions of age (by 10-year age bands), sex and deprivation (by SIMD decile). The dashed line at SMR = 100 represents equivalence in mortality rate between the two populations; that is, if the adjusted death rates were the same in the two populations, the confidence intervals would be expected to overlap the dashed line.

Figure 1 compares mortality rates from 1999 to 2008 between the 410 record-linked “revolving door” patients and the general Scottish population. It shows the standardised mortality ratio (SMR) in the “revolving door” cohort, overall and within age, sex and deprivation subgroups, relative to the general population in Scotland in 2004 [9]. The SMR is defined as the number of deaths observed among “revolving door” patients for every 100 deaths in the general Scottish population. The SMR estimates were adjusted to take account of differences in mortality rate between the two populations due to differences in the distributions of age, sex and deprivation.

The overall SMR for the “revolving door” patient cohort was 333 (95% CI 264 to 415) for every 100 deaths in the general population. In other words, there were around three to four deaths in the “revolving door” cohort for every death in the general population. Among younger “revolving door” patients (<45 years), the SMR is 558 (95% CI 401 to 726), more than double the SMR of patients aged over 45 (relative risk 2.5, 95% CI 1.6 to 3.7), so the death rate is approximately six-fold higher than in the general population. There were no significant differences in SMR between males and females or the most deprived 20% and the least deprived 80%.

### Apparent disappearance

That “revolving door” patients were reducing in number was evident when the first interviews were conducted in 2006 as described by this key informant:

I (interviewer): “…this idea of “revolving door” patients do you think that's a valid one?”

R (respondent): “I might have a couple of years back but I don’t think so much now. The GP contract changed in 2004 and my allocations have literally gone down to zilch so the contract has been great for me. I do have the offenders, my ones that are continually going round the system but in saying that they stay longer with a practice now before they are put off; they are no longer a seven day removal; so it’s working for me.” (Practitioner Services respondent (PS4))

The scale of the reduction in “revolving door” patient numbers became apparent in 2009, (four years after the previously described “revolving door” patient cohort) when no patients meeting the definition of a “revolving door” patient (as defined earlier) were eligible for recruitment into a patient experience study.

**Figure 2:**
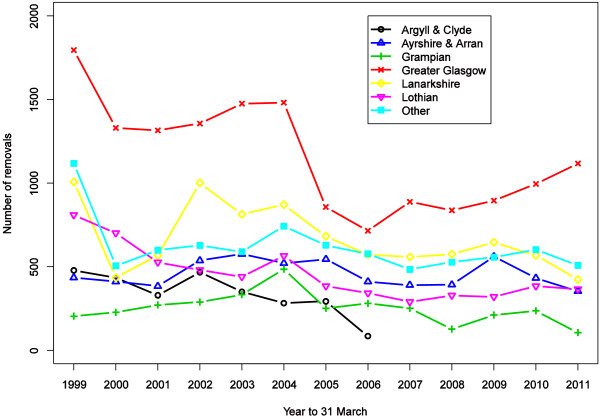
Plot of number of all patient removals due to breakdown of doctor patient relationship or violence, by Scottish Health Board from 1999 to 2011.

Figure 2 plots all removal episodes for reasons of breakdown in doctor patient relationship or violence (excluding geographical removals) by Health Board in Scotland between 1999 and 2011.

Glasgow (and Clyde) demonstrates most removal activity. It is the largest Health Board in Scotland and has the highest proportion of patients living in deprived areas [10].

Table 4 shows the Scottish data for repeat removals from 1999 to 2011. These are calculated within-year so patients who may span a number of years to reach the definition of a “revolving door” patient (including patients with one removal episode per year over four years) are under-represented in these data.

**Table 4:** **Number of repeatedly removed patients by frequency removed within-year from 1999 to 2011 (to end of March) in Scotland**^1^

**Year til March**	**Number of patients removed per number of times**									**As percentage of total removals**
Number of times removed	2	3	**4**	**5**	**6**	**7**	**8**	**9**	**10 +**	
**1999**^2^	264	71	**24**	**9**	**12**	**11**	**6**	**2**	**7**	13.3%
**2000**^3^	90	20	**4**	**3**	**8**	**8**	**8**	**1**	**4**	6.5%
**2001**	149	32	**13**	**12**	**1**	**0**	**0**	**1**	**4**	9.4%
**2002**	256	59	**26**	**7**	**3**	**1**	**1**	**0**	**1**	12.4%
**2003**	147	33	**10**	**2**	**3**	**0**	**0**	**0**	**2**	7.0%
**2004**^4^	159	35	**9**	**5**	**1**	**1**	**3**	**0**	**3**	7.1%
**2005**	154	29	**4**	**6**	**2**	**0**	**0**	**2**	**2**	6.0%
**2006**	102	15	**6**	**3**	**0**	**0**	**0**	**0**	**2**	6.0%
**2007**	118	15	**4**	**0**	**0**	**1**	**0**	**0**	**0**	5.1%
**2008**	106	9	**4**	**2**	**0**	**0**	**0**	**0**	**0**	4.6%
**2009**	121	9	**0**	**1**	**0**	**0**	**0**	**0**	**0**	4.3%
**2010**	74	8	**1**	**0**	**2**	**1**	**0**	**0**	**0**	2.8%
**2011**	95	7	**1**	**1**	**0**	**0**	**0**	**0**	**0**	3.8%

^1^ Data from personal communication: Information Services Division NHS National Services: *Patient removal data including repeat removals from GP lists in Scotland, 1999 to end of March 2011*. 15-11-2011.

^2^ Excludes repeat data for Greater Glasgow, Fife and Dumfries and Galloway Health Board.

^3^ Excludes repeat data for Greater Glasgow and Lanarkshire Health Board.

^4^ 2001-2004 inclusive excludes repeat data for Greater Glasgow Health Board.

Had it been possible to include the repeat removal data about individual patients for Glasgow and Clyde Health Board from 1999 to 2004 the trend downwards may have been steeper.

The professional key informants described three reasons why they thought the generation of “revolving door” patients had reduced so dramatically over time.

The first was the introduction of the treatment of problem drug use and subsequent development of services. A GP respondent gave a bleak description of the early years of the drug injecting epidemic and then how that changed:

“.it really kicked off about 92, 93, a lot of people started appearing, we had no training in it, we didn’t know what to do. GPs didn’t know what to do, there was no hospital base, there was an alcohol service but there wasn’t a drug service and more people were appearing and we didn’t know what to do with them. Over time, some of these patients became so insistent and abusive and demanding of practices that eventually they would, we would try our best with them but they would cross a line. …when we got a drugs service which was effective and people were getting into treatment, and they were being stabilised, then a lot of these patients’ problems disappeared” GP4.

The second reason was the influence external organisations such as the Royal College of General Practitioners and the Health Ombudsmen had, by discouraging GPs from removing patients from their lists.

The third reason was the impact of the 2004 General Medical Services (nGMS) GP contract which changed the way that practices worked and their payment mechanism. This was considered by all key respondents to have had a large positive influence on practice removal activity and the production of “revolving door” patients. The most important aspects were thought to be the non discriminatory tone and accountability for removal decisions that the contract introduced.

A strong theme that emerged from the GP professional key informants was that when “revolving door” patients stopped “revolving” and settled with a practice they remained challenging to work with. There was limited evidence that a very small number of patients was still being removed, but at a much slower rate than previously. This is illustrated by the following quote from a GP interview from 2010. The practice had kept the patient registered for nine months before this removal:

R: “There would be one that I could think of that most recently left with mild learning difficulties and significant mental health…who again had the difficult way of interacting with the staff, out of hours, and inappropriate requests for things that were insoluble…Unfortunately her [relative] verbally, well no physically threatened [an ancillary member of staff], tried to run [them] over; which was something that we couldn’t really tolerate. And so because he drove her here, all the time on a daily basis; generally that it was something we could not sustain. So she was already on a warning for behaviour and she apologised for it; her behaviour about verbally abusing several members of the reception staff at the front door as they left to go home from work. …she crossed the line it was just unacceptable…”GP3.

## Discussion

This study defined a “revolving door” patient as one who was removed four or more times from GP lists in seven years (excluding those removed for having moved out of the practice area). “Revolving door” patients in this cohort had substance misuse problems and a mixture of psychiatric health problems which included opiate dependency, harmful alcohol use, self harm and for some a diagnosis of personality disorder. Physical health problems were important too, along with being a victim of violence. There was a high risk of dying in this cohort, with one in six patients having died by the time the study concluded despite this being a predominantly younger adult age population of patients. There was no link between removal rates and use of health services although patients being admitted to hospital tended to be close to removal dates and drug treatment episodes tended to be close to re-registration dates.

There was a dramatic decline in the number of patients who became “revolving door” patients over the time frame of the study and this was considered to be due to changes in the way the NHS worked with patients. Developments in problem drug use treatment came early and more recently the expansion of community treatment services which included integrated working between GPs, community addiction teams, hospitals and prisons, is thought to have led to further improvements in stability of treatment. Hence it is postulated that patients whose primary reason for becoming a “revolving door” patient was their difficult interaction with GPs about their drug misuse treatment, stopped “revolving”. There was pressure too from professional bodies to reduce patient removals and the final change appears to be the positive ethical, regulatory, and financial climate of the 2004 nGMS GP contract.

The main strength of this study was that it used mixed methods. This allowed us to contextualise data, direct data collection, and highlight strengths and weaknesses of the data sources. It explored and then represented the complexity of the topic of “revolving door” patients and their health issues, and this ranged across disease areas rather than focusing on one clinical aspect. A limitation of the study was the poor quality of the patient removal data from which the patient cohort was derived from. Also all options for imputing the data had some drawbacks, so our sample could not claim to be the whole cohort of Scottish patients removed from 1999 to 2005 (Additional file 1: Appendix 1). The implications of choosing a discrete time frame of 1999 to 2005, which yielded the best available data at the time of data retrieval, was that patients who were just beginning to revolve prior to or after the cut-off dates would have been excluded. Another limitation was that routine NHS health data were extracted from secondary care sources only, as primary care and emergency department data were not available for data linkage with Community Health Index (CHI) records and the available outpatient data poorly recorded ICD10 diagnostic codes.

This was the first study to investigate the topic of “revolving door” patients in general practice and to describe their morbidity and mortality. It attempted to represent and understand the complex health problems of these patients by reporting across disease categories and using qualitative analysis.

This study highlighted too that the number of “revolving door” patients has reduced dramatically in number in Scotland and that this was considered by the professional key informant to be due to a change in the response of the NHS to these patients. Although we cannot prove that a change in NHS response caused the decline in numbers, it seems to us the most likely explanation. This finding mirrors a historical study of two centuries of hospital admissions of “revolving door” psychiatric patients in the USA which concluded that it was the health service response to patients that caused the increase in patients revolving through the inpatient care system [11].

That the “revolving door” patients described in this study had significant morbidity and mortality means the issue should be taken seriously. The evidence presented here should help us reframe the issue to consider that patients who struggle to form or maintain positive doctor-patient relationships may have high levels of complex morbidity and have a greatly raised risk of mortality.

We have proposed that it is the response of the NHS that has altered sufficiently to reduce the number and speed at which patients do “revolve”. If true, this is a positive development. We think treatment and services for dependent drug users should take much of the credit for this, along with the ethical pressure professional bodies have brought to bear. One possibly surprising influence has been the likely impact of the 2004 nGMS contract. This contract has in the past been praised for success in driving up standards in the provision of care for specific aspects of clinical conditions but with an opportunity cost to management of diseases outwith target areas and to the delivery of traditional GP holistic care [12]. We have provided evidence of a beneficial effect for this small number of disadvantaged patients who had complex morbidity and high mortality rates.

## Conclusions

In conclusion, our findings raise two challenges. First, now that “revolving door” patients are remaining registered with practices for much longer, are practices able to provide effective care for these patients? Second, assuming that the NHS is a complex adaptive system, what impact may future changes to the NHS have on these patients’ ability to remain registered in practices and receive good care?

## Competing interests

The authors declare no competing interests.

## Authors’ contribution

AEW undertook the conception, design, and interpretation of the study for her PhD studentship (2004–2011) with PW and KM supervising and advising as PhD supervisors at the University of Glasgow. AEW carried out the analysis of the qualitative data, and guided the statistical analysis of the patient cohort with the supervision of PW and KM. The statistical analysis was conducted by PCDJ. All authors contributed to the writing of the paper. AEW is the guarantor. The study adheres to the RATS guidelines on qualitative research (http://www.biomedcentral.com/ifora/rats). All authors read and approved the final manuscript.

## Pre-publication history

The pre-publication history for this paper can be accessed here:

http://www.biomedcentral.com/1471-2296/13/95/prepub

## Supplementary Material

Additional file 1 **Appendix 1.** Statistical methods. Appendix 2 Using “patient scripts”. Click here for file

Additional file 2 **Appendix 3.** Additional tests comparing “fast”, “medium” and “slow” revolving door patients. Appendix 4 Summary of the 3 samples of “revolving door” patients. Click here for file
