# Supplementary material for: The disappearance of the “revolving door” patient in Scottish general practice: successful policies
Source: BMC Fam Pract. 2012 Oct 4;13:95. doi: 10.1186/1471-2296-13-95 (PMC3528483; doi:10.1186/1471-2296-13-95)
Supplement: Additional file 1 — Appendix 1. Statistical methods. Appendix 2 Using “patient scripts”. [file 1471-2296-13-95-S1.doc]

**Appendix 1: Statistical methods**

*Data cleaning and imputation to reach the ‘revolving door’ cohort*

The ‘revolving door’ cohort was derived from a larger database of all 52,298 removal and re-registration records for all 33,560 patients removed at least once from practices in Scotland from 1999 to 2005. Each record included a patient identifier, date of birth, date of registration or removal, patient’s postcode of residence and GP practice code. All variables were complete except for practice and postcode, which had 325 (0.6%) and 916 (1.8%) missing values, respectively.

Multiple removals or registrations of the same patient at the same practice on the same day were assumed to be duplicates. Duplicate records were removed, leaving 52,251 records on 33,560 patients.

Missing removal and registration records were imputed. A patient with two consecutive removals was assumed to have a missing registration record, and vice versa. To allow the median time on a practice list to be estimated, dates for these imputed records were also imputed. using the pattern of gaps between events. For example, if a patient’s removal dates tended to fall shortly before registration dates but a long time after the preceding removal date (i.e. a tendency for rapid re-registration and relatively long periods on a list), then this information was used in imputing missing dates. Specifically, for each patient missing dates were imputed as the median fractional location of a record date between the preceding and subsequent date its two neighbours. If this median was not available (because there were no complete sequences of three consecutive dates), then the most common removal-to-registration gap across the whole data set, which was zero days, was used to impute the remaining missing dates. Excluding patients with fewer than four removals and those under 16 years of age resulted in the ‘revolving door’ cohort, which consisted of 7,165 records, 962 (13.4%) of which had been imputed, on 555 patients.

*Statistical analysis*

Continuous data were summarised by the mean and standard deviation (SD), except for skewed data where the median and interquartile range (IQR) were used. Between-group differences were tested using Wilcoxon rank sum tests (continuous variables) and Fisher exact tests (categorical variables).

Correlation between numbers of removal episodes and other patterns of health service activity were estimated by Spearman’s ρ statistic and tested using the asymptotic t-distribution approximation.

To investigate possible links between practice events (removals and registrations) and patients’ health service interactions, we used logistic regression to estimate the odds of a health service date falling close to a practice date (that is, in the first or last 25% or the period between practice dates) and test the null hypothesis that practice and health service events were independent (odds = 1). Correlations of multiple events within patients were accounted for using mixed effects logistic regression, with a random effect fitted within each patient. Dates falling on weekends (< 3% of dates) and imputed dates were excluded from this analysis.

The standardised mortality ratio (SMR) was estimated as the number of deaths registered in the ‘revolving door’ cohort from 1999 to 2008 for every 100 deaths registered in the general population of Scotland in 2004 with the same age group (categorised in 10-year bands from 15-24, 25-34, etc), sex and SIMD decile distributions (reference data provided by National Records of Scotland). 2004 is in the middle of the range 1999-2008 and was assumed to be representative of the mortality rate in the general population during this period. The 2009 version of SIMD was used in the reference data, whereas SIMD 2006 was used to record deprivation in the “revolving door” cohort. The 2006 and 2009 SIMD deciles are closely correlated (Pearson’s *r* = 0.97) and in close agreement, with 97% of data zones either remaining the same or changing by a single decile from 2006 to 2009. 95% confidence intervals around the SMR estimates were approximated from 10,000 bootstrap replicates.

All statistical analyses were conducted using the R statistical software version 2.14.0[[1]](#footnote-2).Mixed effects logistic regression models were fitted using the lme4 [[2]](#footnote-3)package for R.

**Appendix 2: Using “patient scripts”**

### Patient profiles

AEW constructed a profile of each patient as a synthesis of 4 sources of data; the information retrieved from the Community Health Index data on patient removals before the data was successfully imputed, the linked Scottish Drug Misuse Database data (SDMR) the linked hospital admissions data, and the linked outpatient attendance data. This profile was viewed as a way of summarising the unwieldy data for each individual patient contained in the linked data sets.

The profiles were then read and re-read and free text coding was applied using the Charmazian grounded theory approach. Data segments were coded according to the themes that emerged. Some codes were determined by the presence or absence of information contained in the profiles, such as coding for presence or absence on the Scottish Drug Misuse database. Others required consideration about deciding boundaries between codes. Clinical coding used knowledge and assumptions from a GP perspective to interrogate the data and characterise the clinical presentations that the patient profiles contained and were applied if the patient had evidence of a condition relating to that code from their hospital admissions or outpatient attendances.

*Patient scripts*

Script theory has been investigated in medical education to help understand and explain clinical reasoning. Clinicians, in their mental processes, formulate a set of “illness scripts”; templates of symptoms, signs and characteristics encountered during their medical training and clinical practice, which they refine with clinical experience. During a clinical encounter the doctor gathers information and without conscious thought “activates” an illness script or number of scripts.

As the analysis proceeded there was an awareness that patients were being categorised in the same way as when receiving information about patients in day to day clinical work; by activating “patient scripts”. These “patient scripts” were typified as the predominant health problems that the patient had and were developed to capture all clinical diagnoses retrievable such that no areas were missed or ignored.

Each category decision was made explicitly using a graded evidence of strong to weak with diagnostic codes used in hospital admission data viewed as the strongest. For example, if a patient had evidence of hospital admissions in their patient profile where a psychiatric diagnostic code was applied then this was viewed as strong evidence of psychiatric illness. An example of weak evidence was having treatment episode(s) recorded on the Scottish Drug Misuse Database (SDMR). A detailed analysis of number of episodes and substances misused was not carried out; so although having SDMR treatment episodes may mean that the patient has a significant substance misuse health problem, it may also mean that the patient had one treatment episode but then went on to become substance misuse free. Treatment services have varied over time and geography in their thresholds for treatment and the substances they offer treatment for too. If however the patient then goes onto have hospital admissions that relate to drug dependency; if they have diagnostic codes for such, or problems that usually directly relate to drug dependency, such as phlebitis or bacterial endocarditis, then the predominant clinical picture is seen as substance misuse. This too replicates the real world use of “patient scripts”; in that the shape of the patient is about the health dominant conditions or issues that doctors create their script about.

PW and KM reviewed 10% of the patient profiles having been furnished with limited information about the coding hierarchy and detail. They achieved 45% agreement using this approach and it was concluded that the difference was because AEW used a more strict application of the level of evidence considerations set out above. The same reviewers reviewed a further 10% of the sample once they received this detailed information about the coding hierarchy and background. They achieved 70% and 60% agreement respectively. The difference was again attributable to degrees of levels of evidence; for example when each of us decided how strong the evidence was to attribute a “substance misuse combined physical illness” script to a patient rather than “drug dependency problems” alone.

1. R Development Core Team (2011). R: A language and environment for statistical computing. R Foundation for Statistical Computing, Vienna, Austria. URL http://www.R-project.org/. [↑](#footnote-ref-2)
2. Douglas Bates, Martin Maechler and Ben Bolker (2011). lme4: Linear mixed-effects models using S4 classes. R package version 0.999375-42. http://CRAN.R-project.org/package=lme4 [↑](#footnote-ref-3)
