# Supplementary material for: The disappearance of the “revolving door” patient in Scottish general practice: successful policies
Source: BMC Fam Pract. 2012 Oct 4;13:95. doi: 10.1186/1471-2296-13-95 (PMC3528483; doi:10.1186/1471-2296-13-95)
Supplement: Additional file 2 — Appendix 3. Additional tests comparing “fast”, “medium” and “slow” revolving door patients. Appendix 4 Summary of the 3 samples of “revolving door” patients. [file 1471-2296-13-95-S2.doc]

**Appendix 3: additional tests comparing “fast”, “medium” and “slow” revolving door patients**

| **Table 1.** **Presence in SMR01 dataset (general and acute inpatient and day case), years recorded in SMR01, number of admissions per year, number of irregular discharges per year.** | | | | | | | |
| --- | --- | --- | --- | --- | --- | --- | --- |
|  |  | Total | Median days on GP list | | | P[[1]](#footnote-2) slow vs rest | P Fast vs medium |
|  |  |  | Fast (0-100) | Medium (101-180) | Slow (181+) |  |  |
| In SMR01 | N_OBS_ (N_MISSING_) No Yes | 410 (0) 59 (14.4%) 351 (85.6%) | 272 (0) 35 (12.9%) 237 (87.1%) | 77 (0) 13 (16.9%) 64 (83.1%) | 61 (0) 11 (18.0%) 50 (82.0%) | 0.428 | 0.354 |
| Length of time in SMR01 (years) | N_OBS_ (N_MISSING_) Mean (SD) Median (IQR) [Range] | 410 (0) 21.8 (5.9) 22.7 (16.9, 28.1) [7.0, 28.6] | 272 (0) 21.9 (6.0) 22.9 (17.1, 28.5) [7.0, 28.6] | 77 (0) 21.2 (5.7) 21.1 (16.4, 26.5) [8.7, 28.6] | 61 (0) 22.0 (5.8) 22.5 (16.8, 28.6) [9.9, 28.6] | 0.826 | 0.321 |
| No of admissions per year | N_OBS_ (N_MISSING_) Mean (SD) Median (IQR) [Range] | 410 (0) 0.8 (1.2) 0.4 (0.1, 1.0) [0.0, 14.9] | 272 (0) 0.8 (1.3) 0.4 (0.2, 1.0) [0.0, 14.9] | 77 (0) 0.9 (1.2) 0.5 (0.1, 1.1) [0.0, 6.7] | 61 (0) 0.9 (1.2) 0.5 (0.2, 0.9) [0.0, 5.8] | 0.901 | 0.801 |
| No of irregular discharges per year | N_OBS_ (N_MISSING_) Mean (SD) Median (IQR) [Range] | 410 (0) 0.09 (0.18) 0.03 (0.00, 0.12) [0.00, 1.61] | 272 (0) 0.08 (0.16) 0.03 (0.00, 0.11) [0.00, 1.61] | 77 (0) 0.10 (0.16) 0.00 (0.00, 0.15) [0.00, 0.73] | 61 (0) 0.14 (0.27) 0.03 (0.00, 0.12) [0.00, 1.29] | 0.433 | 0.856 |
| Any irregular discharges | N_OBS_ (N_MISSING_) No Yes | 410 (0) 200 (48.8%) 210 (51.2%) | 272 (0) 130 (47.8%) 142 (52.2%) | 77 (0) 42 (54.5%) 35 (45.5%) | 61 (0) 28 (45.9%) 33 (54.1%) | 0.678 | 0.305 |

| **Table 2.  Presence in SMR00 dataset (outpatients attendance), years recorded in SMR00, number of outpatients appointments per year, number of missed outpatients appointments per year.** | | | | | | | |
| --- | --- | --- | --- | --- | --- | --- | --- |
|  |  | Total | Median days on GP list | | | P  slow vs rest | P Fast vs medium |
|  |  |  | fast (0-100) | medium (101-180) | slow (181+) |  |  |
| In SMR00 | N_OBS_ (N_MISSING_) No Yes | 410 (0) 6 (1.5%) 404 (98.5%) | 272 (0) 6 (2.2%) 266 (97.8%) | 77 (0) 0 (0.0%) 77 (100.0%) | 61 (0) 0 (0.0%) 61 (100.0%) | 0.598 | 0.345 |
| Length of time in SMR00 (years) | N_OBS_ (N_MISSING_) Mean (SD) Median (IQR) [Range] | 410 (0) 13.0 (1.9) 14.0 (13.0, 14.0) [4.7, 14.0] | 272 (0) 12.9 (2.1) 14.0 (12.5, 14.0) [4.7, 14.0] | 77 (0) 13.1 (1.7) 14.0 (13.1, 14.0) [6.2, 14.0] | 61 (0) 13.3 (1.4) 14.0 (14.0, 14.0) [8.6, 14.0] | 0.128 | 0.876 |
| No of appointments per year | N_OBS_ (N_MISSING_) Mean (SD) Median (IQR) [Range] | 410 (0) 2.1 (2.5) 1.2 (0.6, 2.8) [0.0, 17.8] | 272 (0) 2.0 (2.5) 1.2 (0.6, 2.7) [0.0, 17.8] | 77 (0) 2.4 (2.7) 1.2 (0.6, 3.4) [0.0, 12.6] | 61 (0) 2.2 (2.5) 1.3 (0.7, 2.4) [0.1, 10.3] | 0.770 | 0.464 |
| No of missed appointments per year | N_OBS_ (N_MISSING_) Mean (SD) Median (IQR) [Range] | 410 (0) 0.8 (1.0) 0.6 (0.2, 1.0) [0.0, 10.4] | 272 (0) 0.8 (1.0) 0.6 (0.3, 1.0) [0.0, 10.4] | 77 (0) 0.9 (1.0) 0.5 (0.2, 1.1) [0.0, 4.5] | 61 (0) 0.9 (1.0) 0.6 (0.2, 1.1) [0.0, 4.0] | 0.828 | 0.661 |
| Any missed appointments | N_OBS_ (N_MISSING_) No Yes | 410 (0) 31 (7.6%) 379 (92.4%) | 272 (0) 21 (7.7%) 251 (92.3%) | 77 (0) 5 (6.5%) 72 (93.5%) | 61 (0) 5 (8.2%) 56 (91.8%) | 0.795 | 1.000 |

| **Table 3.  Number of prison or court referrals per decade and number of patients with any prison or court referrals** | | | | | | | |
| --- | --- | --- | --- | --- | --- | --- | --- |
|  |  | Total | Median days on GP list | | | P slow vs rest | P Fast vs medium |
|  |  |  | fast (0-100) | medium (101-180) | slow (181+) |  |  |
| No of prison or court referrals per decade | N_OBS_ (N_MISSING_) Mean (SD) Median (IQR) [Range] | 410 (0) 0.5 (1.8) 0.0 (0.0, 0.0) [0.0, 27.1] | 272 (0) 0.4 (1.0) 0.0 (0.0, 0.0) [0.0, 7.9] | 77 (0) 0.6 (1.5) 0.0 (0.0, 0.7) [0.0, 10.0] | 61 (0) 1.0 (3.7) 0.0 (0.0, 0.0) [0.0, 27.1] | 0.592 | 0.088 |
| Any prison or court referrals | N_OBS_ (N_MISSING_) No Yes | 410 (0) 329 (80.2%) 81 (19.8%) | 272 (0) 224 (82.4%) 48 (17.6%) | 77 (0) 57 (74.0%) 20 (26.0%) | 61 (0) 48 (78.7%) 13 (21.3%) | 0.729 | 0.106 |

| **Table 4.  N (%) patients with at least one hospital admission for each listed diagnosis category** | | | | | | | |
| --- | --- | --- | --- | --- | --- | --- | --- |
|  |  | Total | Median days on GP list | | | P slow vs rest | P Fast vs medium |
|  |  |  | fast (0-100) | medium (101-180) | slow (181+) |  |  |
| Injury | N_OBS_ (N_MISSING_) No Yes | 410 (0) 136 (33.2%) 274 (66.8%) | 272 (0) 89 (32.7%) 183 (67.3%) | 77 (0) 26 (33.8%) 51 (66.2%) | 61 (0) 21 (34.4%) 40 (65.6%) | 0.883 | 0.891 |
| Learning impairment | N_OBS_ (N_MISSING_) No Yes | 410 (0) 406 (99.0%) 4 (1.0%) | 272 (0) 270 (99.3%) 2 (0.7%) | 77 (0) 76 (98.7%) 1 (1.3%) | 61 (0) 60 (98.4%) 1 (1.6%) | 0.476 | 0.528 |
| Poisoning | N_OBS_ (N_MISSING_) No Yes | 410 (0) 195 (47.6%) 215 (52.4%) | 272 (0) 128 (47.1%) 144 (52.9%) | 77 (0) 38 (49.4%) 39 (50.6%) | 61 (0) 29 (47.5%) 32 (52.5%) | 1.000 | 0.796 |
| Substance misuse | N_OBS_ (N_MISSING_) No Yes | 410 (0) 132 (32.2%) 278 (67.8%) | 272 (0) 88 (32.4%) 184 (67.6%) | 77 (0) 29 (37.7%) 48 (62.3%) | 61 (0) 15 (24.6%) 46 (75.4%) | 0.184 | 0.413 |
| Diagnostic label not applied[[2]](#footnote-3) | N_OBS_ (N_MISSING_) No Yes | 410 (0) 89 (21.7%) 321 (78.3%) | 272 (0) 53 (19.5%) 219 (80.5%) | 77 (0) 19 (24.7%) 58 (75.3%) | 61 (0) 17 (27.9%) 44 (72.1%) | 0.238 | 0.340 |
| Psychiatric illness | N_OBS_ (N_MISSING_) No Yes | 410 (0) 253 (61.7%) 157 (38.3%) | 272 (0) 168 (61.8%) 104 (38.2%) | 77 (0) 46 (59.7%) 31 (40.3%) | 61 (0) 39 (63.9%) 22 (36.1%) | 0.776 | 0.791 |
| Physical illness | N_OBS_ (N_MISSING_) No Yes | 410 (0) 90 (22.0%) 320 (78.0%) | 272 (0) 61 (22.4%) 211 (77.6%) | 77 (0) 15 (19.5%) 62 (80.5%) | 61 (0) 14 (23.0%) 47 (77.0%) | 0.867 | 0.641 |
| Intervention or procedure | N_OBS_ (N_MISSING_) No Yes | 410 (0) 208 (50.7%) 202 (49.3%) | 272 (0) 137 (50.4%) 135 (49.6%) | 77 (0) 41 (53.2%) 36 (46.8%) | 61 (0) 30 (49.2%) 31 (50.8%) | 0.890 | 0.699 |

| **Table 5.  N (%) patients with at least one hospital admission for each highlighted condition** | | | | | | | |
| --- | --- | --- | --- | --- | --- | --- | --- |
|  |  | Total | Median days on GP list | | | P slow vs rest | P Fast vs medium |
|  |  |  | fast (0-100) | medium (101-180) | slow (181+) |  |  |
| Alcohol misuse | N_OBS_ (N_MISSING_) No Yes | 410 (0) 257 (62.7%) 153 (37.3%) | 272 (0) 174 (64.0%) 98 (36.0%) | 77 (0) 51 (66.2%) 26 (33.8%) | 61 (0) 32 (52.5%) 29 (47.5%) | 0.085 | 0.788 |
| Drug misuse | N_OBS_ (N_MISSING_) No Yes | 410 (0) 177 (43.2%) 233 (56.8%) | 272 (0) 114 (41.9%) 158 (58.1%) | 77 (0) 36 (46.8%) 41 (53.2%) | 61 (0) 27 (44.3%) 34 (55.7%) | 0.889 | 0.515 |
| Negative behaviour recorded | N_OBS_ (N_MISSING_) No Yes | 410 (0) 390 (95.1%) 20 (4.9%) | 272 (0) 259 (95.2%) 13 (4.8%) | 77 (0) 73 (94.8%) 4 (5.2%) | 61 (0) 58 (95.1%) 3 (4.9%) | 1.000 | 1.000 |
| Personality disorder diagnosis | N_OBS_ (N_MISSING_) No Yes | 410 (0) 335 (81.7%) 75 (18.3%) | 272 (0) 223 (82.0%) 49 (18.0%) | 77 (0) 60 (77.9%) 17 (22.1%) | 61 (0) 52 (85.2%) 9 (14.8%) | 0.590 | 0.414 |
| Physical consequences of alcohol misuse | N_OBS_ (N_MISSING_) No Yes | 410 (0) 364 (88.8%) 46 (11.2%) | 272 (0) 241 (88.6%) 31 (11.4%) | 77 (0) 70 (90.9%) 7 (9.1%) | 61 (0) 53 (86.9%) 8 (13.1%) | 0.659 | 0.681 |
| Self harm | N_OBS_ (N_MISSING_) No Yes | 410 (0) 213 (52.0%) 197 (48.0%) | 272 (0) 138 (50.7%) 134 (49.3%) | 77 (0) 45 (58.4%) 32 (41.6%) | 61 (0) 30 (49.2%) 31 (50.8%) | 0.678 | 0.247 |
| Violence victim | N_OBS_ (N_MISSING_) No Yes | 410 (0) 249 (60.7%) 161 (39.3%) | 272 (0) 161 (59.2%) 111 (40.8%) | 77 (0) 53 (68.8%) 24 (31.2%) | 61 (0) 35 (57.4%) 26 (42.6%) | 0.572 | 0.145 |

**Appendix 4: summary of the 3 samples of “revolving door” patients**

| Background to the patient sample and comparison of patient characteristics | | | | |
| --- | --- | --- | --- | --- |
|  |  | patients included in morbidity and mortality linkage | patients included in qualitative analysis | patients included in demographic description |
| Sex | N_OBS_ (N_MISSING_) N (%) Male | 410 (0) 281 (68.5%) | 351 (0) 239 (68.1%) | 555 (0) 371 (66.8%) |
| Age (years) at first removal | N_OBS_ (N_MISSING_) Mean (SD) | 410 (0) 34 (13) | 351 (0) 34 (13) | 555 (0) 34 (13) |
| SIMD decile at first removal | N_OBS_ (N_MISSING_) Median (IQR) | 409 (1) 9.0 (7.0, 10.0) | 350 (1) 9.0 (7.0, 10.0) | 409 (146) 9.0 (7.0, 10.0) |
| Median days on GP list | N_OBS_ (N_MISSING_) Median (IQR) | 410 (0) 49 (20, 132) | 351 (0) 37 (17, 94) | 555 (0) 86 (26, 174) |
| Reason for patients excluded |  | Earliest definition excluded patients with median days on list greater than 366 days and other “slow” patients | Definition developed to include current fast and medium definitions but excluded slow patients. | Removed fewer than 4 times from GP lists over 7 years only |

1. P-values are from Wilcoxon rank sum tests (continuous variables) and Fisher exact tests (categorical variables). [↑](#footnote-ref-2)
2. Symptom or presentation description without a diagnosis [↑](#footnote-ref-3)
